# The effect of modulating platelet reactive oxygen species by the addition of antioxidants to prevent clearance of cold-stored platelets

**DOI:** 10.1016/j.htct.2024.09.2479

**Published:** 2024-10-18

**Authors:** Rufeng Xie, Yiming Yang, Xueyu Jiang, Li Gao, Juan Sun, Jie Yang

**Affiliations:** Blood Engineering Laboratory, Shanghai Blood Center, Shanghai, China

**Keywords:** Cold-stored platelets, Reactive oxygen species (ROS), N-acetylcysteine (NAC), Clearance

## Abstract

**Background:**

It is known that the rapid clearance of cold-stored platelets is attributed to various storage lesions, including an abnormal increase in reactive oxygen species when platelets are exposed to cold temperatures. As an antioxidant, N-acetylcysteine exhibits some significant effects on scavenging various reactive oxygen species and inhibiting cell damage and apoptosis.

**Aims:**

This study aimed to investigate the effects of N-acetylcysteine on reducing reactive oxygen species production and protecting cold-stored platelets from phagocytosis and clearance, and to determine the optimal concentration of N-acetylcysteine.

**Methods:**

Platelet concentrates were divided into three groups: room-temperature-stored platelets, cold-stored platelets, and cold-stored platelets with the addition of different concentrations of N-acetylcysteine. After five days of storage, reactive oxygen species production, lipid peroxidation levels, activation marker expressions, GPIb/ɑ desialylation with exposure of glycan residues and other quality parameters of platelets were measured and compared between the groups. Phagocytosis of platelets was detected by phorbol 12-myristate 13-acetate-activated THP-1 or Hep G2 cells. Moreover, the recovery of infused platelets was measured in severe combined immunodeficient mice at different timepoints.

**Results:**

After 5 days of storage, cytoplasmic reactive oxygen species significantly increased in chilled compared to non-chilled platelets; they were notably reduced with the addition of N-acetylcysteine, particularly at a concentration of 5 mM. Compared with chilled platelets, the P-selectin and phosphatidylserine expressions, as well as exposure of GPIb/ɑ glycan residues, were significantly reduced with 5 mM of N-acetylcysteine. Phagocytosis of platelets by THP-1 or Hep G2 cells was significantly lower in 5 mM of N-acetylcysteine compared to cold-stored platelets without N-acetylcysteine.

**Conclusions:**

This study demonstrated correlations between reactive oxygen species production and their pro-oxidant effects on platelet clearance after cold storage. The addition of N-acetylcysteine at an appropriate concentration do not only protects chilled platelets from storage lesions caused by reactive oxygen species overproduction but also prevents platelet phagocytosis *in vitro* and clearance *in vivo*, thereby extending circulating time.

## Introduction

As is well known, platelet transfusion is the main management of thrombocytopenia, the crucial guarantee for surgery accompanied by severe hemorrhage and a powerful adjunct to other treatments. However, despite improvements in platelet storage, there remains an unmet demand for platelet products due to the constantly increasing need for platelet treatments.^1^^,^^2^ For decades, platelets for clinical transfusion have been stored on agitators at room temperature (22 ± 2 °C) worldwide with the expiration period for platelets being limited to 5 to 7 days due to the risk of contamination by bacteria and other pathogens.^3^, ^4^, ^5^ Cold storage is the best possible alternative strategy to overcome the limitations of room temperature storage. Cold storage of platelets has the potential advantage of reducing the risk of contamination while extending the shelf time.^6^ In addition, cold-stored platelets (CSP) appear to be healthier, as they maintain a stable pH of 7.2 to 7.4, exhibit enhanced aggregation, and demonstrate stronger hemostatic effects.^7^, ^8^, ^9^, ^10^

However, cold storage temperature (4 ± 2 °C) results in platelet damage, including shape changes, rearrangement of the cytoskeleton, degradation of microfilament proteins, decreased recovery rate of hypotonic shock response, and even spontaneous aggregation.^11^, ^12^, ^13^, ^14^ Furthermore, CSP are rapidly removed from blood circulation with a significantly lower recovery compared to that of room temperature-stored platelets (RTP).^15^^,^^16^ While the mechanism of cold-induced lesions on platelets is still unclear, the clearance process was proposed in early 2003.^17^ This process involves the clustering of glycoprotein Ib subunits, the exposure of β-N-acetylglucosamine (β-GlcNAc) and galactose residues caused by desialylation, and then recognition and clearance by macrophages and hepatocytes *in vivo*.^18^^,^^19^ Therefore, inhibiting the rapid clearance of CSP *in vivo* and improving recovery after infusion are crucial for the clinical application of CSP.

The remarkable feature of platelets, which sets them apart from other cells, is the absence of a cell nucleus. Therefore, mitochondrial function and viability play a crucial role in the platelet metabolism and survival.^20^^,^^21^ Previous researches have found that cold storage impairs mitochondrial function, leading to decreased mitochondrial trans-membrane potential promoting GPIb/α clustering.^22^^,^^23^ More importantly, it was revealed that reactive oxygen species (ROS), the crucial signal regulatory molecules, were continuously generated by mitochondria during the cold storage of platelets.^24^ In the physiological state, the dynamic balance between intracellular ROS production and clearance is maintained, which keeps ROS at low levels in platelets. Once platelets are activated, more ROS are generated. High levels of ROS could further enhance platelet activation and impact their function. Meanwhile, mitochondrial function becomes abnormal, and platelets generate excessive ROS, leading to the oxidation of protein sulfhydryl groups and unsaturated fatty acids, ultimately resulting in platelet lesions.^25^ The addition of ROS scavengers, such as N-acetylcysteine (NAC),^26^ cytochrome C^27^ and resveratrol,^28^ could significantly reduce the ROS levels of refrigerated platelets and improve the *in vitro* viability of platelets in cold storage. Especially NAC, which is a potent antioxidant, has core properties that include the inhibition of ROS-induced cellular damage and apoptosis, as well as cytoprotective functions both *in vitro* and *in vivo*.^29^^,^^30^ Thus, NAC is a potential candidate for an additive solution to maintain platelet characteristics during cold storage.

This study investigated the correlation between cytoplasmic and mitochondrial ROS and their pro-oxidant effect on platelet clearance following cold storage. The optimal concentration of the antioxidant NAC was selected based on its corrective effect on excessive ROS production and platelet activation, thus this study aims to provide practical guidance for cold-stored platelet products.

## Materials and methods

### Platelet preparation and study design

Pooled human platelets were randomly collected from the Shanghai Blood Center and processed using the buffy-coat-derived platelet method. Buffy-coat-derived platelet units from four ABO identical healthy donors were pooled and aliquoted within 8 h after collection. The day of collection was defined as Day 0. In the study design, three groups were established: 1) room-temperature-stored platelets (RTP) - platelets stored at 20 to 24 °C with continuous agitation; 2) Cold-stored platelets (CSP) - platelets stored at 4 to 6 °C without agitation; 3) CSP-NAC: stored as CSP with the addition of different concentrations (1 mM, 5 mM, 25 mM) of NAC (Sigma, USA). Pooled platelets from the groups were analyzed at different timepoints. After sampling, the platelet pools were immediately returned to their storage conditions.

### Ethics statement

This study was approved by the Ethics Committee of the Shanghai Blood Center (Permit number: SBC-IRB-2021–03). All donors signed an informed consent form for their blood samples to be used in this scientific research. The animal study was conducted in strict accordance with the guidelines of the Institutional Animal Care and Use Committee of the Chinese Association for Laboratory Animal Sciences. All surgeries were performed using diethyl ether, and every attempt was made to minimize suffering.

### Platelet quality parameter measurements

The platelets from each group described above were sampled aseptically on Day 1 and Day 5 of storage. The platelet count and mean platelet volume (MPV) were measured by a hematology analyzer (Mindray BC-5150, China). The pH was measured using a pH electrode (SevenCompact™ S230, Mettler Toledo, Switzerland).

### Analysis of reactive oxygen species production

The production of ROS was measured using CellROX® oxidative stress reagents (Invitrogen, USA), which are fluorogenic probes designed to accurately measure ROS in live cells. According to the instructions, mitochondrial ROS (mito-ROS) were measured using CellROX® Green Reagent, which is a DNA dye that binds to DNA in platelet mitochondria. Cytoplasmic ROS (cyto-ROS), localized in the cytoplasm, were measured using CellROX™ Deep Red. The CellROX® Reagent was added at a final concentration of 5 μM to the platelets of each group and incubated for 30 min at 37 °C. ROS levels, expressed as mean fluorescence intensity (MFI), were measured by flow cytometer (CytoFLEX, Beckman Coulter, USA).

To investigate the impact of rewarming on ROS production, the five-day-stored RTP, CSP and CSP-NAC were rewarmed to 37 °C for 30 min. Subsequently, the levels of cyto-ROS and mito-ROS were measured by fluorescent activated cell sorting (FACS). The increase in ROS levels was reported as the ratio of ROS MFI after platelet rewarming *versus* those without rewarming.

### Lipid peroxidation assay

Lipid peroxidation was assessed using the Image-iT® Lipid Peroxidation Kit (Invitrogen, USA), which relies on the BODIPY® 581⁄591 C11 reagent and is a sensitive fluorescent reporter for lipid peroxidation. According to the instructions, the platelets of each group were incubated with Image-iT® Lipid Peroxidation Sensor (10 µM) for 30 min at 37 °C, then washed three times. MFIs at 590 nm (red) and 510 nm (green) were measured by FACS and the ratios of the MFIs at 590 nm to 510 nm were used to quantify lipid peroxidation in platelets.

### Detection of platelet activation markers

Platelet activation was assessed by measuring the P-selectin expression (CD62P) using FACS. Exposure of phosphatidylserine on the platelet surface, as an activatation and apoptosis marker, was measured by annexin V binding. Briefly, the platelets from each group were diluted to 1 × 10^6^ platelets/mL in Tyrode's buffer and were incubated with anti-human CD62P-PE monoclonal antibodies (BD Biosciences, USA) or annexin V- fluorescein isothiocyanate (FITC) monoclonal antibodies (Invitrogen, USA) for 15 min at room temperature, and according isotype controls were also included in the experiment. P-selectin and phosphatidylserine expressions were reported as percentages of CD62P positive and Annexin V positive cells, respectively.

### Thromboelastography platelet mapping

Platelet coagulation function was assessed using thromboelastography (TEG 5000, Haemonetics Corporation, USA) enabling real-time monitoring for prompt results and offering data on time to clot (R), clotting velocity (α angle), clot strength and other functional parameters. According to the manufacturer's guidelines, the thromboelastography platelet mapping analysis was performed using platelet concentrates from each group stored for five days, and the R time and the maximal amplitude (MA) values of platelets from each group were compared.

### Glycoprotein GPIb/ɑ expression and glycan exposure

The frequency of the glycoprotein GPIb/ɑ on platelets from each group was determined as the MFI on binding to the anti-human CD42b monoclonal antibodies (BD Biosciences, USA) by FACS. The GPIb/α glycan residues, β-galactose (β-Gal) and β-N-acetylglucosamine (β-GlcNAc), were detected by their binding to specific fluorescein-labeled lectins (Vector Laboratories, USA), *e.g.*, ricinus communis agglutinin (RCA) I binds to β-Gal and succinylated wheat germ agglutinin (sWGA) binds to β-GlcNAc. Exposed β-Gal and β-GlcNAc were measured by FACS and reported as the MFI of RCA I and sWGA, respectively.

### *In vitro* phagocytosis

*In vitro* phagocytosis was achieved and modified^23^ with the THP-1 or Hep G2 cell lines (Cell Bank/Stem Cell Bank, Shanghai Institute of Biochemistry and Cell Biology, China) being used as macrophages or hepatocytes. Briefly, monocytic THP-1 cells were induced by phorbol 12-myristate 13-acetate (100 ng/mL; Sigma, USA) for 48 h, and then activated by lipopolysaccharide (100 ng/mL; Sigma, USA) for 24 h. These adherent cells were identified as activated macrophages. Platelets from each group (RTP, CSP and CSP-NAC5) were incubated with carboxyfluorescein succinimidyl ester (CFSE - 5 μM; Invitrogen, USA) for 15 min at 37 °C. CFSE-labeled platelets were incubated with activated macrophages for 1 h at 37 °C. Non-adherent and extracellularly adhered platelets were removed by three washes with 1 % ethylenediaminetetraacetic acid-phosphate buffered saline. Subsequently, the macrophages were detached by treating with TrypLE Express (Invitrogen, USA) for five minutes at 37 °C. Cells were collected and stained with anti-human CD41-PE monoclonal antibodies (BD Biosciences, USA) and analyzed by flow cytometry. Macrophages were selected based on their forward/side scatter characteristics and CFSE/CD41 staining. CFSE^+^/CD41^−^ events in macrophages were quantified relative to the total CD41-negative macrophage population. The percentage of CFSE^+^/CD41^−^ cells was defined as the rate of platelet phagocytosis by macrophages. As a similar method, the phagocytosis rate of platelets by hepatocytes was measured using human hepatocellular carcinoma (Hep G2) cells without *in vitro* induction/activation.

### Analysis of *in vivo* platelet recovery

Severe combined immunodeficient mice (SCID) mice (male, 6–8 weeks old, 18–20 g; Shanghai Laboratory Animal Center of the Chinese Academy of Science, China) were used for human platelet transfusions.^31^ Five-day-stored human platelets (2 × 10^9^ platelets/mice) of the different groups (RTP, CSP and CSP-NAC5) were individually transferred into SCID mice (five mice/group) via the tail vein. Retro-orbital blood specimens were collected and labeled with anti-human CD41-PE monoclonal antibodies at different timepoints after the transfusion. Platelet recovery was analyzed by flow cytometry. First, test platelets were selected based on a log-scale forward scatter/side scatter gate, in accordance with their varying sizes compared to mouse red blood cells. Then, the percentage of CD41-PE platelets in the total tested platelets was used to calculate the recovery of human platelets. Data were normalized to 100 % for Time-30 min, which was analyzed at 30 min after the human platelet transfusion.

### Statistical analysis

Results were analyzed using GraphPad Prism 9.0 software (GraphPad Software, San Diego, CA, USA), and the data are presented as the mean ± standard error of the mean (SEM). Linear regressions were calculated based on the least square differences method and presented as R^2^. To compare differences among multiple groups, 95 % confidence intervals (95 % CI) are included. One-way or two-way ANOVA was performed followed by Tukey's multiple comparisons test. A paired *t*-test was used to calculate differences between two groups. A *p*-value <0.05 was defined as statistically significant.

## Results

### N-acetylcysteine can partially maintain cold-stored platelets hematological parameter values

The platelet count (cells/mL), pH level and mean platelet volume (MPV) of RTP, CSP and CSP-NAC were evaluated on Day 1 and 5 of storage. As shown in Table 1, the platelet count decreased during storage in all groups with cold storage resulting in a greater reduction in the platelet count compared to conventional storage at room temperature. The addition of NAC did not improve this condition, and there were no significant differences between the groups. The MPV values of platelets in each group had increased after being stored for five days. The addition of a high dosage of NAC (25 mM) significantly elevated the MPV value. As expected, the pH levels of RTP were lower than those of all cold-stored platelets after five days of storage. Although the pH of each group decreased with the storage time due to metabolic accumulation, this reduction could be effectively prevented by a low concentration of NAC (1 or 5 mM). These data indicate that the addition of an appropriate dose of NAC can maintain an acceptable pH and quality of platelets after being cold-stored for five days.

**Table 1:** Evaluation of hematological parameters in the different groups.

		Days of platelet storage	
Parameter	Group	Day 1 Mean ± SEM *n* = 15	Day 5 Mean ± SEM *n* = 15
Platelet count (x 10^6^/mL)	RTP	559.3 ± 20.94	531.6 ± 15 46
	CSP	532.3 ± 15.28	497.9 ± 10.87
	CSP-NAC1	525.3 ± 17.84	505.4 ± 15.05
	CSP-NAC5	524.3 ± 13.30	503.4 ± 12.48
	CSP-NAC25	535.7 ± 8.93	506.0 ± 13.74
pH	RTP	7.23 ± 0.019^a^	7.08 ± 0.025
	CSP	7.29 ± 0.037^a^	7.14 ± 0.027
	CSP-NAC1	7.31± 0.020	7.20 ± 0.033^b^
	CSP-NACS	7.29 ± 0.013	7.19 ± 0.029^b^
	CSP-NAC25	7.25±0.025^a^	7.1l ± 0.033
MPV	RTP	7.74 ± 0.26	8. 21 ± 0.27
	CSP	8.10 ± 0.35	8.59 ± 0.27
	CSP-NAC1	8.20 ± 0.36	8.66 ± 0.26
	CSP-NAC5	8.34 ± 0.37	8.82 ± 0.27
	CSP-NAC25	9.03 ± 0.35^a^	9.76 ± 0.25^b^

NAC, N-acetylcysteine; RTP, room-temperature-stored platelets; CSP, cold-stored platelets; CSP-NAC, CSP with the addition of 1 mM (CSP-NAC1), 5 mM (CSP-NAC5) and 25 mM (CSP-NAC25) of NAC; SEM, standard error of the mean; MPV, mean platelet volume.

a *p*-value <0.05 comparing between each group at different timepoints.

b *p*-value <0.05 comparing between different groups and the RTP group at same timepoint.

### N-acetylcysteine can effectively prevent increased production of reactive oxygen species and inhibit lipid peroxidation

**Figure 1:**
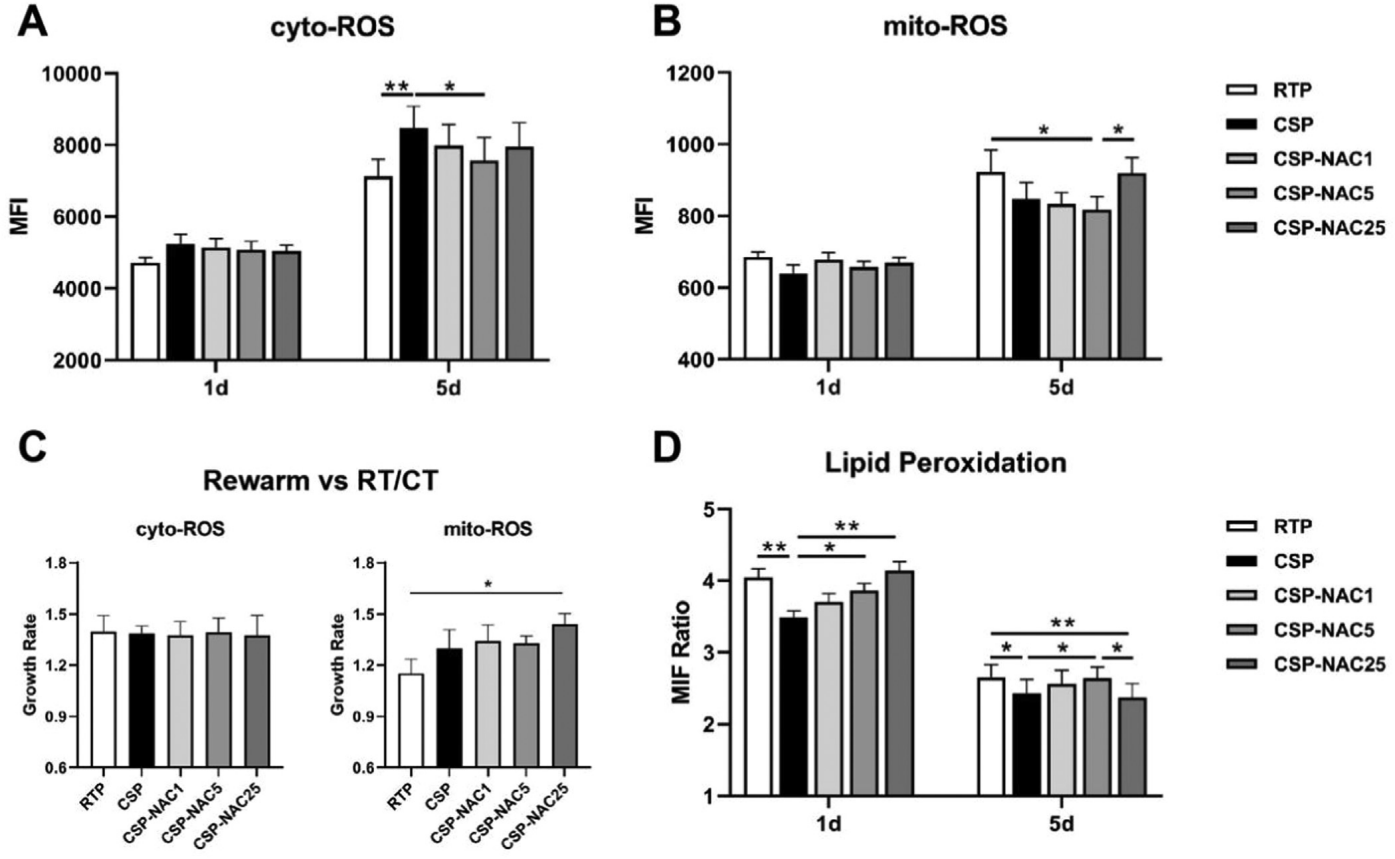
Antioxidant N-acetylcysteine (NAC) prevents increases in the production of cytoplasmic (cyto)-reactive oxygen species (ROS) and mitochondrial (mito)-ROS and inhibits lipid peroxidation in cold-stored platelets (A) Cyto-ROS production of room-temperature-stored platelets (RTP), cold-stored platelets (CSP) and CSP with the addition of NAC (CSP-NAC) was measured using fluorescent activated cell sorting (FACS) with CellROX™ Deep Red and reported as mean fluorescence intensity (MFI). (B) Mito-ROS of the three aforementioned groups was measured by FACS with CellROX® Green Reagent, and reported as MFI. (C) After rewarming platelets of 5-day-stored RTP, CSP and CSP-NAC to 37 °C for 30 min, the MFI of cyto-ROS and mito-ROS were measured by FACS. The effect of rewarming on ROS production is expressed by the rate of increase in ROS levels, which is the ratio of ROS-MFI after platelet rewarming *versus* those without rewarming. (D) Lipid peroxidation was measured using the Image-iT® Lipid Peroxidation Kit. MFIs at 590 nm (red) and 510 nm (green) for each group of platelets were measured by fluorescent activated cell sorting (FACS) and the ratios of the MFIs at 590 nm to 510 nm were used to quantify lipid peroxidation in platelets, inversely proportional to the level of lipid peroxidation. The results are shown as means ± standard error of the mean (*n* = 6). *p-value <0.05, **p-value <0.01, ***p-value <0.001, compared between the groups.

The levels of cytoplasmic ROS (cyto-ROS) and mitochondrial ROS (mito-ROS) in CSP treated with or without NAC were analyzed and compared in order to determine the effect of the antioxidant in reducing ROS production. The FACS results showed that the cyto-ROS production in RTP and especially in CSP had increased after five days of storage, with the cyto-ROS level of CSP being significantly higher than that of RTP. The increase in cyto-ROS levels of CSP was effectively prevented by the addition of NAC, particularly with the addition of 5 mM of NAC (Figure 1A). In contrast to cyto-ROS production, the mito-ROS level in CSP was significantly lower than that in RTP after five days of storage, although both showed an increase in mito-ROS levels. With the addition of a low concentration of NAC (1 or 5 mM), CSP maintained a low level of mito-ROS, while high dosages of NAC (25 mM) seemed to promote mito-ROS production in CSP (Figure 1B).

To determine the effect of rewarming on ROS production, platelets stored for five days from each group were rewarmed, with the increased ROS levels being reported as the ratio of ROS MFI after platelets were rewarmed *versus* those without rewarming. As shown in Figure 1C, both cyto- and mito-ROS production of platelets of all groups increased after rewarming. The increase in mito-ROS levels in CSP was markedly higher than those in RTP, indicating that rewarming induced more mito-ROS production in CSP. The addition of low concentrations of NAC (1 or 5 mM) did not influence the increase in mito-ROS levels during rewarming in CSP. Upon rewarming, CSP with 25 mM of NAC exhibited a significant increase, even an overproduction, of mito-ROS due to the excessively high rate (approximately 1.5 times higher).

Lipid peroxidation is the degradation of lipids that occurs as a result of oxidative damage and serves as a valuable marker for oxidative stress. Thus, the levels of lipid peroxidation in CSP with and without NAC were also determined and compared. On Day 1, there was a higher level of lipid peroxidation in CSP than in RTP. The addition of NAC decreased lipid peroxidation in CSP, as evidenced by increased ratios in CSP-NAC. This correction was positively correlated with the dose of NAC. After five days of storage, all groups of platelets showed an increase in lipid peroxidation, and the inhibitory effect of NAC on lipid peroxidation was very limited.

These data indicate that the antioxidant NAC, when administered at an appropriate dose, could effectively prevent the increased production of ROS, independent of cyto-ROS or mito-ROS, and inhibit lipid peroxidation during the early stages of storage. This helped reduce oxidative stress damage in CSP and improved their storage quality.

### N-acetylcysteine can effectively inhibit the activation of cold-stored platelets, reduce phosphatidylserine exposure and maintain coagulation function

**Figure 2:**
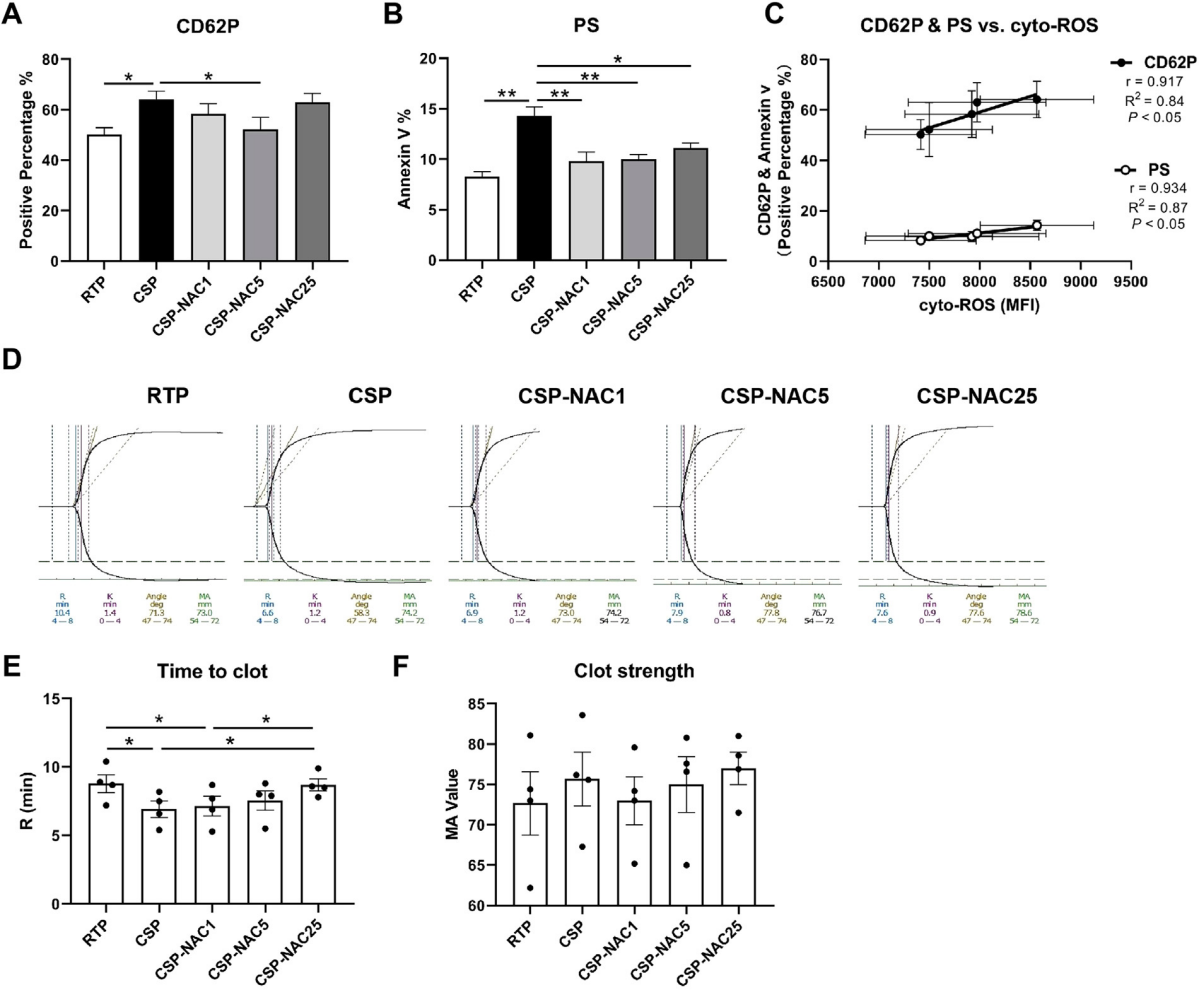
The addition of N-acetylcysteine (NAC) effectively inhibits platelet activation, reduces phosphatidylserine exposure and maintains coagulation function (A) The membrane P-selectin expression of room-temperature-stored platelets (RTP), cold-stored platelets (CSP) and CSP with the addition of NAC (CSP-NAC) after five days is indicated as positive percentage of binding CD62P antibodies by fluorescent activated cell sorting (FACS). (B) The exposure of phosphatidylserine of 5-day-stored RTP, CSP and CSP-NAC is indicated as positive percentage of Annexin V by FACS. (C) Correlation and linear regression of the CD62P and phosphatidylserine expressions with the cytoplasmic (cyto)-reactive oxygen species (ROS) production after five days of storage. The results are shown as means ± standard error of the mean (*n* = 6). (D) The thromboelastography platelet mapping (TPM) analysis was performed using the 5-day-stored platelets in each group. (E) The time to clot and (F) clot strength of platelets of each group were compared. The results are shown as means ± standard error of the mean (*n* = 4). *p-value <0.05, **p-value <0.01, ***p-value <0.001, compared between the groups.

To determine the effect of adding antioxidants to shield platelets from cold-induced damage, platelet activation was compared in the RTP, CSP and CSP-NAC groups by analyzing the membrane expression of P-selectin (CD62P) and exposed phosphatidylserine (Annexin V). Different concentrations of NAC were used to determine the optimal dosage of antioxidants. The FACS results showed that CD62P expression of CSP was significantly higher compared to that of RTP after five days of storage. By adding different doses of NAC, the levels of CD62P on CSP were all reduced by varying degrees. A NAC concentration of 5 mM exhibited the most potent inhibition of CD62P expression on CSP. Both lesser (1 mM) and higher (25 mM) concentrations showed limited suppression. Meanwhile, phosphatidylserine exposure was notably increased in CSP, while RTP showed low phosphatidylserine exposure after five days of storage. Once NAC was added, regardless of the concentration (low or high), the level of phosphatidylserine exposure in CSP decreased significantly, approaching that of RTP. As expected, there was a significant correlation between the expressions of CD62P and phosphatidylserine with the levels of cyto-ROS production in platelets stored for five days (Figure 2C), which indicates a direct correlation between cyto-ROS and platelet activation.

Further, some key parameters related to the coagulation function of RTP, CSP and CSP-NAC were measured using thromboelastography platelet mapping (Figure 2D), and the time to clot and clot strength of platelets from each group were compared. The results of the thromboelastometry profile showed that after five days of storage, the coagulation time (R time) of CSP was significantly shorter than that of RTP stored for 5 days. After the addition of NAC, the coagulation time increased, indicating that the time taken for clotting is directly proportional to the concentration of NAC added. When the NAC concentration reached 25 mM, the coagulation time was almost no different from RTP (Figure 2E). In addition, the MA of platelets from each group was analyzed, which represents the maximal strength of the clot and indicates the degree of platelet aggregation. As shown in Figure 2F, there was greater aggregation in CSP than in RTP, and this aggregation was partially relieved upon the addition of NAC, although there were no significant differences between the MA values of the three groups.

Taken together, these data strongly suggest that the addition of an appropriate dose of NAC could effectively inhibit platelet activation, reduce phosphatidylserine exposure, and maintain coagulation function, thereby reducing cold-storage-induced damage to platelets.

### N-acetylcysteine can protect glycoprotein GPIb/α and decrease exposure of β-Gal and β-GlcNAc residues due to cold-storage lesions

**Figure 3:**
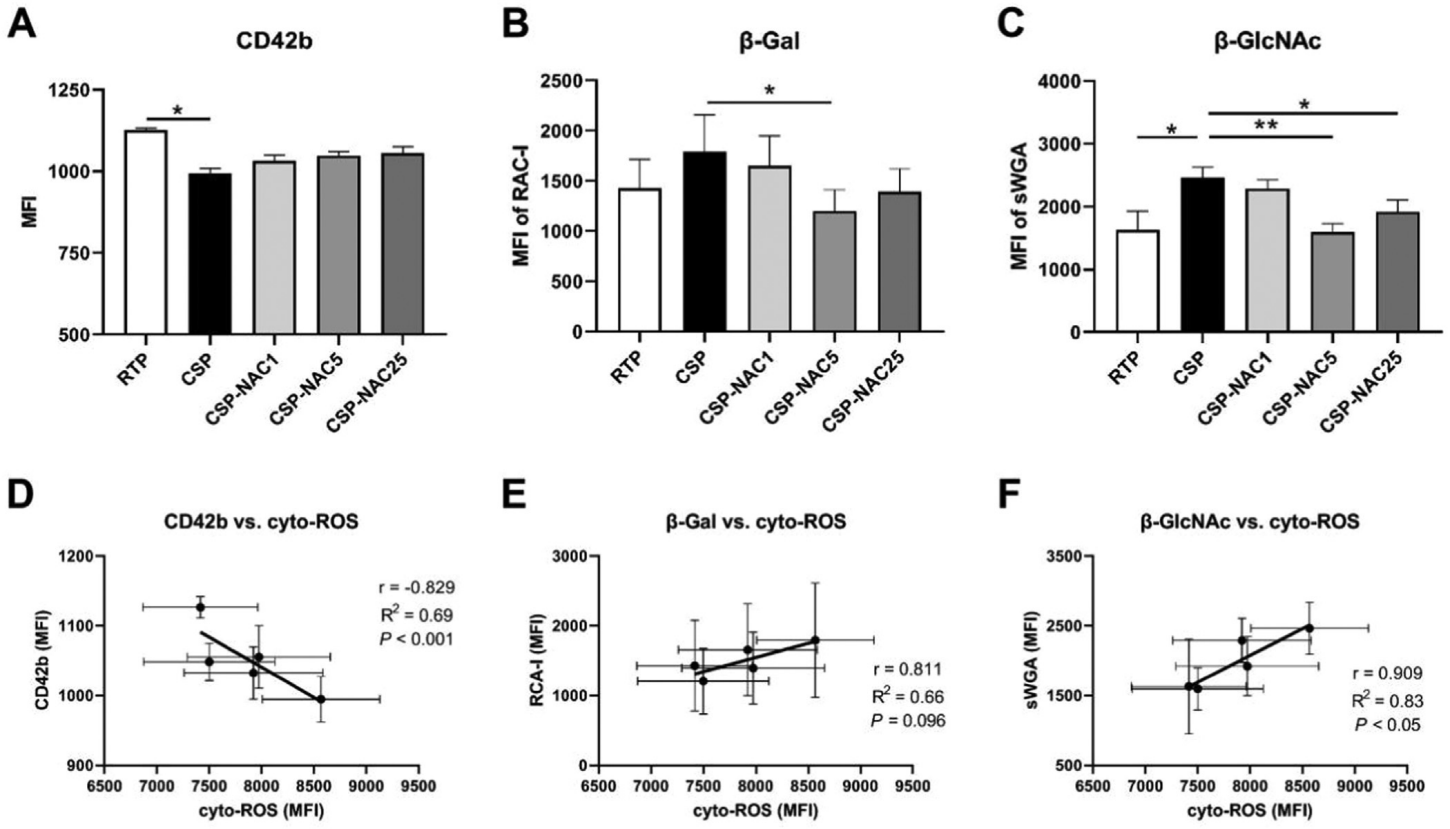
N-acetylcysteine (NAC) protects glycoprotein GPIb/α and deceases exposure of β-Gal and β-GlcNAc due to cold-storage lesions (A) The GPIb/ɑ level of room-temperature-stored platelets (RTP), cold-stored platelets (CSP) and CSP with the addition of NAC (CSP-NAC) stored for five days is indicated as the mean fluorescence intensity (MFI) of binding to CD42b antibodies. (B) The exposure of β-Gal and (C) β-GlcNAc on 5-day-stored RTP, CSP and CSP-NAC are indicated as MFIs of RAC I and sWGA binding probes assessed by fluorescent activated cell sorting (FACS), respectively. The results are shown as means ± standard error of the mean (*n* = 6). *p-value <0.05, **p-value <0.01, ***p-value <0.001, compared between the groups. (D) Correlation and linear regression between CD42b expression and the levels of cyto-ROS production after five days of storage. (E) Correlation and linear regression between β-Gal expression and the levels of cyto-ROS production after five days of storage. (F) Correlation and linear regression between β-GlcNAc expression and the levels of cyto-ROS after five days of storage.

Following refrigeration, clustering of glycoprotein GPIb/ɑ and exposed glycan residues initiates recognition by phagocytosis *in vitro* and *in vivo*.^32^^,^^33^ Thus, the platelet expressions of GPIb/α and its residues in RTP, CSP and CSP-NAC were quantified and compared to confirm the protective effect of NAC on GPIb/α subunits. The GPIb/ɑ frequency of platelets from each group is indicated as the MFI by FACS due to CD42b antibody binding. Contrary to RTP, CSP maintained a low frequency of GPIb/ɑ after five days. The addition of NAC appeared to increase the level of GPIb/ɑ in CSP, although the difference was not significant. The level of GPIb/ɑ also increased with increasing concentrations of NAC, but the impact of this was very limited. Meanwhile, exposed β-galactose (β-Gal) and β-N-acetylglucosamine (β-GlcNAc) on GPIb/α, which play an important role in increased lectin recognition, were detectable through their binding to specific lectins. The FACS results indicated that both β-Gal and β-GlcNAc levels of platelets showed a significant increase after five days of cold storage. After adding various concentrations of NAC, the exposure of β-Gal and β-GlcNAc on CSP decreased. A concentration of 5 mM NAC appeared to be the optimal dosage by providing the best protection against the exposure of β-Gal and β-GlcNAc. Furthermore, the correlation between cyto-ROS production in stored platelets and GPIb/ɑ expression, as well as the exposure of glycan residues, was analyzed (Figure 3D-E). There was a greater negative correlation between CD42b expression and cyto-ROS formation, and a positive correlation between β-Gal/β-GlcNAc exposure and cyto-ROS production.

These results suggest that the addition of NAC could protect glycoprotein GPIb/α and decrease the exposure of β-Gal and β-GlcNAc due to cold-storage lesions, thereby preventing CSP from being recognized and cleared.

### N-acetylcysteine can efficiently prevent cold-stored platelets from being phagocytized by macrophages and hepatocytes

**Figure 4:**
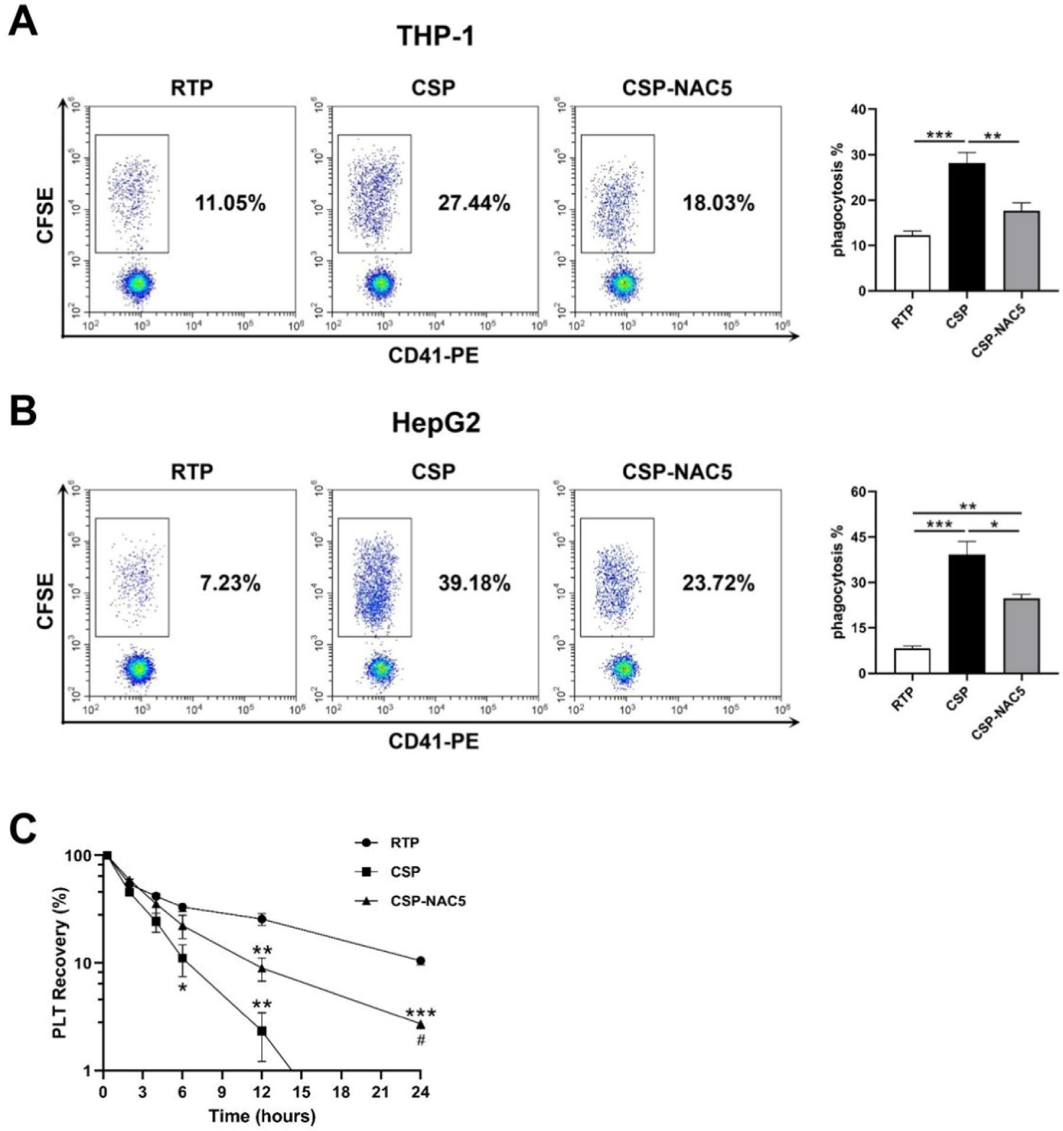
The additional of N-acetylcysteine (NAC) significantly prevents cold-storage platelets from recognition and clearance *in vitro* and *in vivo* (A) Activated THP-1 cells were used to analyze macrophage-dependent phagocytosis after being co-cultured with 5-day-stored room-temperature-stored platelets (RTP), cold-stored platelets (CSP) and CSP with the addition of 5 mM NAC (CSP-NAC5). (B) Hep G2 cells were used to analyze hepatocyte-dependent phagocytosis after co-culturing with 5-day-stored RTP, CSP and CSP-NAC5. The percentage of CFSE^+^/CD41^−^ cells was measured by fluorescent activated cell sorting (FACS) and defined as the phagocytosis rate for each sample. The results are shown as means ± standard error of the mean (*n* = 6). *p-value <0.05, **p-value <0.01, ***p-value <0.001, compared between the groups. (C) Five-day-stored RTP, CSP and CSP-NAC5 (2 × 10^9^ platelets/mice) were transferred into SCID mice (5 mice/group) via the tail vein. Retro-orbital blood specimens were collected and labeled using anti-human CD41-PE monoclonal antibodies at different timepoints after the transfusion. The percentage of CD41^+^ cells was measured by FACS and defined as platelet recovery rate. The results are shown as means ± standard error of the mean (*n* = 5). *p-value <0.05, **p-value <0.01, ***p-value <0.001, compared between the indicated groups and the RTP group at the same timepoint; #*p*-value <0.05, compared between the indicated groups and CSP group at the same timepoint.

It has been reported that cold-induced damage to platelets leads to the reorganization of membrane glycoproteins, which results in recognition and clearance mediated by macrophage integrins (αMβ2 integrin) or hepatocyte Ashwell-Morell receptors.^17^, ^18^, ^19^ Activated THP-1 cells and Hep G2 cells were used to determine whether adding NAC could prevent macrophage-dependent and hepatocyte-dependent CSP phagocytosis. Phagocytosis was analyzed after co-culturing with RTP, CSP and CSP-NAC. A NAC concentration of 5 mM was chosen because it appeared that it did not stimulate ROS production and inhibits CSP activation, which was shown to be sufficient to aid in the storage of CSP. The results depicted in Figure 4A indicate that cold storage induced severe platelet damage, leading to a significant increase in macrophage-dependent phagocytosis, while the phagocytosis of RTP remained at low levels. The addition of the antioxidant NAC to CSP efficiently inhibited CSP internalization mediated by macrophages, leading to a decrease in the phagocytosis rate. Similarly, the ingestion of platelets by Hep G2 cells was significantly lower in CSP-NAC5 than in CSP, even though fewer RTP were ingested (Figure 4B).

These data confirm that oxidative stress is the primary mediator of platelet damage, leading to phagocytosis by macrophages and hepatocytes. The additional NAC can significantly improve cold-induced storage damage and prevent CSP from being recognized and cleared *in vitro*.

### N-acetylcysteine can significantly prevent clearance and prolong the lifespan of CSP *in vivo*

To determine whether adding NAC could prevent *in vivo* platelet clearance, RTP, CSP and CSP-NAC5 were transfused into SCID mice with the recovery of human platelets being measured at different timepoints (Figure 4C). Compared with RTP, CSP stored for five days were cleared faster and more efficiently by SCID mice. A significant reduction in the recovery was observed as early as 6 h after the infusion, with <2 % of CSP detected after 12 h of circulation. The addition of 5 mM of NAC to the CSP resulted in a partial extension of the lifespan of CSP *in vivo*; until 12 h after administration, approximately 10 % were circulating *in vivo*, although significantly less compared to RTP. CSP with 5 mM of NAC were detected in nearly 2 % of the samples for as long as 24 h after transfusion, even though the CSP without NAC had almost completely been cleared.

These data demonstrate that ROS play a significant role in the cold-storage damage of platelets, and that the addition of NAC at an appropriate concentration can effectively prevent such damage during storage, thereby extending the circulating time of transfused platelets *in vivo*.

## Discussion

As is well known, there are several potential benefits of cold-stored platelets, including the reduction of bacterial proliferation, preservation of platelet metabolic stores, and increased platelet hemostatic efficacy. Unfortunately, the rapid clearance of refrigerated platelets have been attributed to various cold storage lesions, involving an abnormal increase in ROS. This further leads to a short circulation time and low survival of CSP after transfusion in patients, which caused the abandonment of CSP in clinical applications.^34^^,^^35^ Thus, this study investigated the correlation between ROS and their pro-oxidant effects on platelet clearance following cold storage. The optimal concentration of the antioxidant NAC was chosen according to its corrective effect on excessive ROS production and platelet over-activation, which is expected to provide practical guidance for the application of cold-stored platelet products.

Consistent with several studies that have already shown the increasing levels of ROS production during storage,^23^^,^^28^^,^^36^ this study further confirmed that cyto-ROS production in five-day-stored CSP was markedly higher than in RTP and evidenced a direct correlation between cyto-ROS and the activation and desialylation of platelets. It was found that the activation markers, CD62P and phosphatidylserine, on 5-day-stored CSP were significantly higher compared to those on 5-day-stored RTP. The expressions of these activation markers were positively correlated with cyto-ROS production. Additionally, the results of this study indicated that platelets with higher levels of cyto-ROS exhibited lower expressions of GPIb/α, while showing increased exposure of β-Gal and β-GlcNAc. This ultimately resulted in the platelets being more readily recognized and phagocytized. This finding was further confirmed by the results of the *in vitro* phagocytosis assay and *in vivo* recovery. Fortunately, the addition of NAC at an appropriate concentration significantly suppresses the overproduction of cyto-ROS caused by cold damage, preventing CSP activation and desialylation, thereby protecting CSP from being recognized and cleared.

As platelets are non-nucleated cells, mitochondrial function and viability are particularly important for platelet metabolism and survival.^21^ Thus, the changes in mito-ROS in CSP after five days of storage were analyzed, and the impact of the addition of NAC on mito-ROS was confirmed in the current study. In contrast to the high levels of cyto-ROS in CSP, mito-ROS remained at low levels throughout the storage period due to the reduced metabolic demand of platelets during cold storage. Furthermore, it was observed that platelets with good aggregation responses and short clotting times also exhibited excellent mitochondrial function. These data suggest that a lower level of basal mito-ROS production during cold storage better preserves platelet viability and function than room temperature storage. Meanwhile, CSP preserve the ability to generate an oxidative burst when challenged with rewarming or an agonist, which is crucial for further activation of the coagulation cascade. Excitingly, the addition of NAC at an appropriate concentration helped maintain CSP with low mito-ROS levels and did not affect their oxidative burst.

Recently, various approaches have been attempted to minimize the platelet activation effects of cold temperature storage in order to maintain platelet circulation capacity. One approach involved using temperature cycling of 4 °C for 12 h and 37 °C for 30 min during cold storage.^37^^,^^38^ This method intermittently increases the temperature before irreversible injury occurs to refrigerated platelets. Platelets treated with temperature cycling showed a significantly higher recovery in SCID mice compared to refrigerated platelets. However, their recovery in humans was still unsatisfactory. Another method is to add inhibitors, such as p38MAPK^39^ and endothelium-derived inhibitors,^40^ to the platelet storage media in order to limit platelet activation. However, none of them have been accepted for general application in transfusion. Based on ROS overproduction during platelet activation, various ROS scavengers were selected as supplements during platelet cold storage. It was confirmed that they significantly reduced the ROS levels of CSP and improved their *in vitro* viability.^36^ Among the numerous antioxidants, NAC stands out due to its core properties, which include the inhibition of ROS-induced cellular damage and apoptosis, as well as its *in vitro* and *in vivo* cytoprotective function.^41^

NAC is a thiol compound with the ability to scavenge ROS, which inhibits ROS-induced cellular damage and apoptosis; it is used safely as an anti-platelet compound. The antioxidant capacity of NAC and its positive effect on physiological storage parameters have been proven in various studies.^26^^,^^29^^,^^30^^,^^41^ Yong Gon Cho's group indicated that the addition of 50 mM NAC protected refrigerated concentrates during long-term storage, retaining the integrity of the platelets.^29^^,^^30^ In the present study, we found that increased cyto-ROS production associated with cold storage could be corrected *in vitro* by a lower dosage (5 mM) of NAC. Moreover, a high concentration of NAC may facilitate the formation of mito-ROS and induce platelet activation more readily after five days of cold storage. These differences seem to be related to the type of platelet products with varying platelet concentrations (apheresis platelets *versus* buffy-coat-derived platelets), the storage containers (tubes sealed with paraffin laboratory film *versus* gas-permeable bags), and the criteria for concentration selection (the expression of CD62P after two days of cold storage *versus* the ROS production after five days of cold storage). A study by Cancelas et al. suggested that adding 1 mM of NAC to platelet additive-plasma storage solution could prevent ROS production and correct the macrophage-dependent and -independent clearance of platelets.^23^ Considering the overall investigations and results, the concentration of NAC used must consider the concentration in platelet products. A low concentration of NAC might be safer and more conducive to retaining platelet functional characteristics during long-term cold storage.

Recently, the U.S. Food and Drug Administration (FDA) published guidance on “Alternative Procedures for Cold-Stored Platelets Intended for the Treatment of Active Bleeding when Conventional Platelets Are Not Available or Their Use Is Not Practical” (https://www.fda.gov/media/169714/download). This guidance provides recommendations for blood establishments in the manufacture and labeling of CSP, indicates available data on the quality and use of CSP, and discusses the need for additional data on the efficacy of CSP. Undoubtedly, research on CSP for transfusion is advancing and further studies are anticipated to discover improved methods that prevent damage during cold storage while maintaining the beneficial features of cold-stored platelets.

A limitation of this study is the small number of replicates in some experiments; the sample size needs to be increased to support further clinical studies. Additionally, platelets were stored for only five days due to the storage period limitation of gas-permeable storage bags in the current study. Research on the effects of NAC on CSP with a longer storage period (>5 days) might be considered in the future. The mechanisms involved in ROS protecting cold-stored platelets from phagocytosis also need to be further studied.

## Conclusion

This study demonstrated that a high level of cyto-ROS induced by cold storage was closely related to CSP activation, GPIb/ɑ clustering and desialylation. These factors are crucial contributors to the recognition and clearance of CSP both *in vitro* and *in vivo*. Additionally, low mito-ROS production in CSP helped preserve platelet energy stores. The addition of NAC at an appropriate concentration significantly reduces the levels of platelet cyto-ROS and mito-ROS. This suggests that NAC might help preserve the potential benefits of CSP, and more importantly, reduce CSP recognition and phagocytosis by cells, thereby protecting them from clearance after transfusion.

## Conflicts of interest

The authors certify that they have no affiliation with or financial involvement in any organization or entity with a direct financial interest in the subject matter or materials discussed in this manuscript.
